# A web-based mouse-tracking task for early perceptual language processing

**DOI:** 10.3758/s13428-025-02827-8

**Published:** 2025-10-10

**Authors:** Holger Mitterer

**Affiliations:** 1 Department of Cognitive Science, Faculty of Media and Knowledge Sciences, University of Malta, Msida, 2080 MSD Malta; 2 Hanyang Institute for Phonetics and Cognitive Science, Seoul, South Korea

**Keywords:** Mouse tracking, Prosody, Spoken-word recognition

## Abstract

The study of language processing requires data from a wide range of languages but also data that are free from demand characteristics and meta-linguistic strategies. While eye-tracking has been successfully used to address the later issue, pragmatically, eye-tracking is often difficult to achieve with less well-studied languages. Therefore, the current paper presents a web-based mouse-tracking task that generates data that seem to reflect early perceptual processes similar to eye-tracking but which can be performed remotely. The task uses a set-up similar to early video games to entice participants to use language input as early as possible. The data presented here replicate an earlier eye-tracking study focusing on how reduced words are recognized. Fillers from the same study are also used, which show that the paradigm also reflects predictive semantic processing. It is concluded that the paradigm can be used to investigate lexical access, prosodic processing, and predictive semantic processing.

Two trends in the study of language processing are to some extent incompatible. On the one hand, there is the realization that languages differ in non-trivial ways (Evans & Levinson, 2009), so that language research needs to tackle a variety of different languages instead of focusing on Indo-European languages, which currently dominate the literature (Collart, 2024; Kidd & Garcia, 2022). On the other hand, there is the trend towards more technically advanced methods of measuring language processing that are less influenced by meta-linguistic task demands and decision processes, such as electroencephalography (EEG) (Grimaldi et al., 2023) and eye-tracking (Reinisch & Mitterer, 2022). It is uncontroversial that both goals are worth pursuing. However, pursuing one of these goals makes it difficult to pursue the other as well, since most labs with expensive equipment are “surrounded” by speakers of Indo-European languages. While it is possible to find native speakers of other languages in these “surroundings,” there are usually too few to satisfy the sample size constraints for psycholinguistic experiments (Brysbaert & Stevens, 2018).

Therefore, the current paper investigates whether a mouse-tracking paradigm previously used to investigate prosodic processing (Mitterer et al., 2024) can also be used to investigate early stages of spoken-word recognition as well as predictive semantic processing. Critically, the mouse-tracking experiment is implemented in a web-based setting, making it possible for the research to travel to the location in which a language is spoken without the researcher travelling. As a test of the paradigm, the current study attempts to replicate the results of an eye-tracking study on the effects of segment deletion (Mitterer & Reinisch, 2015).

Mouse-tracking has long been considered a possible alternative to eye-tracking in language research (Spivey et al., 2005). Spivey et al. (2005) argued that mouse-tracking is an interesting converging method to eye-tracking, since eye movements tend to be ballistic, while hand/limb movements are more continuously adjusted to the task goals. In their task, participants were presented with two visual targets at the top of the screen and had to select one of them with the mouse cursor based on the auditory input. The main manipulation in Spivey et al. (2005) was whether the target object (e.g., a picture) was accompanied by a phonological competitor (a pickle) or an unrelated word (e.g., a candy), and the results showed that participants moved the mouse towards the target faster if the other picture was not a phonological competitor.

However, lab-based mouse-tracking often relies on making changes to the cursor speed (Schoemann et al., 2021). This means that mouse-tracking is not easily available in a web-based setting, since JavaScript explicitly excludes (for good reason)^1^ any tempering with the mouse setting. Moreover, it is always a challenge to keep participants engaged in a web-based experiment, with many potential distractions around them.

This challenge was tackled by Mitterer et al. (2024) who used mouse-tracking in a web-based setting and were successful in measuring early processing of prosodic information. In order to compensate for the lack of control over the cursor speed, Mitterer et al. (2024) devised a task based loosely on the early computer game “Galaga”. In this game, the player moves a spaceship in the horizontal direction at the bottom of the screen. At the same time, objects are “falling down” from the top of the screen, while also moving somewhat unpredictably left and right, and the user has to shoot or collect one of the objects by clicking with the mouse when the spaceship is right under the target object (see Fig. 1). By making the two objects move downwards at a constant rate but also moving unpredictably in the horizontal dimension, this task was apparently successful in engaging participants to make an early choice for one of the targets in order to track its horizontal movements. This game-like design also follows the idea to gamify studies that are run in a web-based setting (Hartshorne et al., 2019).

**Fig. 1:**
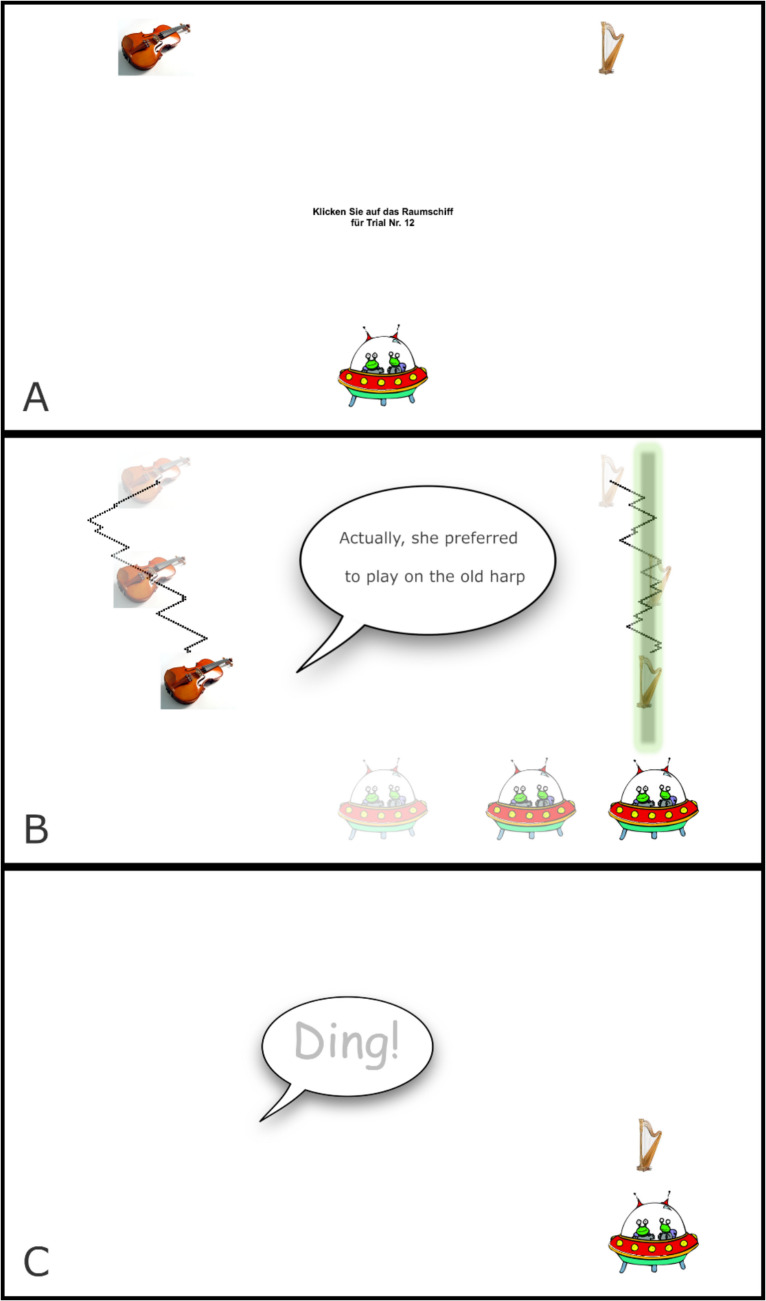
Screen examples for the start routine (**A**), the animation routine (**B**), and the feedback routine (**C**). In panel **B**, the black dots show a possible path of the objects. The objects in their position at the beginning of the screen (mostly transparent), half-way through the trial (half transparent), and when the participant clicked (opaque) to activate the tractor beam. The speech bubble in panel **C** indicates the sound being played as correct feedback.

The dependent variable used in this task by Mitterer et al. (2024) was called “decision time” and referred to the last time that participants moved towards the target. This was based on a similar mouse-tracking study in a laboratory setting by Roettger and Franke (2019) focusing on the use of intonation in language processing, a topic for which there is a long research tradition using eye-tracking (for an overview, see Reinisch & Mitterer, 2022). Roettger and Franke found that participants turn faster towards the target if an intonational cue (the German *verum* accent in reply to a yes/no question) cues the target before the name of the target is uttered. Mitterer et al. (2024) replicated this finding in a web-based mouse-tracking task and extended it to show, first, that it can also be triggered by segmental information, and second, that it is necessary to show that effects apparently triggered by the prosodic properties of the utterance are not simply caused by covariant learning.

While this indicates that the paradigm can be used to investigate prosodic processing, the purpose of the current study is to show that it is useful for other purposes as well. The data to be shown here indicate that the paradigm can also be used to investigate semantic predictive processing as well as how lexical access is affected by variation in the input signal—two foundational issues in psycholinguistic research.

To test the viability of the paradigm for these issues, we performed a conceptual replication of the study by Mitterer and Reinisch (2015). They had studied the effect of deletion of the initial segments /Ɂ/ and /h/ in German words such as *Ampel* /Ɂampəl/ and *Handtuch* /hant:ux/ (English ‘traffic light’ and ‘towel’) on spoken-word recognition. The theoretical impetus for that study was whether the orthographic coding of /h/ in *Handtuch* but not /Ɂ/ in *Ampel* impacts the effects of reduction, as would be predicted by accounts that orthography–phonology mismatches slow down spoken word recognition (Ziegler & Ferrand, 1998). That is, the deletion of /h/ but not of /Ɂ/ creates a mismatch with the orthographic form, because there is no grapheme for /Ɂ/ in German.^2^ Nevertheless, words written as vowel-initial (as *Ampel*) are produced with an initial glottal stop in their canonical pronunciation (Kohler, 1990). It follows that the deletion of /h/ should be more disruptive, because this creates not only a non-optimal input, but also a mismatch with the orthographic form.

The /h/- and glottal stop-initial words occurred in relatively natural conversational sentences with other markers for typical reductions (e.g., German *haben,* English ‘to have’, produced as *ham*). The sentence also provided semantic cues, and the visual display contained the target, a semantic competitor, and an unrelated distractor (e.g., in the case of the target *apple*, the sentence was *In the afternoon, he sometimes likes to eat an apple*, and the semantic competitor was *cake*). The efficiency of word recognition was assessed in a visual-world eye-tracking paradigm, with the instruction to click on one of the objects if it was mentioned in the sentence. To lower the demand characteristics to click on the object that most resembles what is heard, one quadrant of the screen was left empty, with the instruction to click there if none of the three objects displayed matched the sentence. To make this a viable option, there were 48 filler trials, for half of which none of the three objects on the screen was mentioned in the sentence.

The results showed that words were recognized slower if the initial segment was missing, but participants did click on the target objects, which shows that they did recognize the words despite the deleted initial segment. More importantly, these reduction costs were not influenced by the orthographic coding of the deleted segment but were similar for /h/ and the glottal stop. Moreover, the filler trials contained, next to the target, a semantic competitor that also fit the sentence and an unrelated distractor. In the current paradigm, there are only two images on the screen, so we can make use of that to test whether predictive processing is reflected in the mouse-tracking record. To achieve this, the study varied which objects were used as the competitor. Mitterer and Reinisch (2015) had used three objects on the screen, and for the filler trials, one of those was a semantic competitor that would also fit the sentence frame and one that was unrelated. For instance, in a sentence such as “Tenerife offers the … great training opportunities”, the target was *climber*, the competitor was *surfer*, and the unrelated distractor was *pasta*. In the current study, for half of the filler trials, the semantic competitor was used as the second object, and for the other half, the unrelated distractor. This was implemented as a within-item predictor by varying the competitor objects (competitor or unrelated distractor) for a given item over participants. Mitterer and Reinisch (2015) found that listeners looked more at the target and the competitor than at the distractor. A conceptual replication of this result would be that participants go for the target earlier when the other object is the unrelated distractor than when it is the semantic competitor.

To summarize, the current study uses a mouse-tracking paradigm in a web-based setting, and tests whether effects on lexical access and predictive processing are reflected in the mouse-tracking data. Figures 1 and 2 show the basic set-up of the task with three main routines used to run it. For the start routine (Fig. 1, panel A), two objects appear at the top of the screen and a spaceship at the bottom. To normalize the mouse position at the start of the trial, participants have to click on the spaceship to start the trial. This leads to the animation routine during which the two objects fall down with additional wiggling in the horizontal direction. The black dots show a possible path for the objects and the objects at the start, the middle, and the end of the routine with increasing opacity. At the same time, the participants hear a sentence (*Eigentlich spielt sie lieber auf der alten Harfe,* see Fig. 1 for a translation) that contains the name of one of the objects (here: the harp). The participants were asked to move the spaceship, which is initially in the middle of the screen, under the target object. They then have the choice between catching the falling object with the spaceship or activate a “tractor beam” (the greenish bar in Fig. 1B), which is only becomes visible on a mouse click and then draws the object to the spaceship faster than it would fall. When participants catch the object (or the objects reach their lowest possible point), the feedback routine started. The feedback routine showed the target object at the lowest possible point, and if the spaceship was under that point (as in Fig. 1C), a “bling” sound was played; otherwise, a buzzer-type of sound was played.

**Fig. 2:**
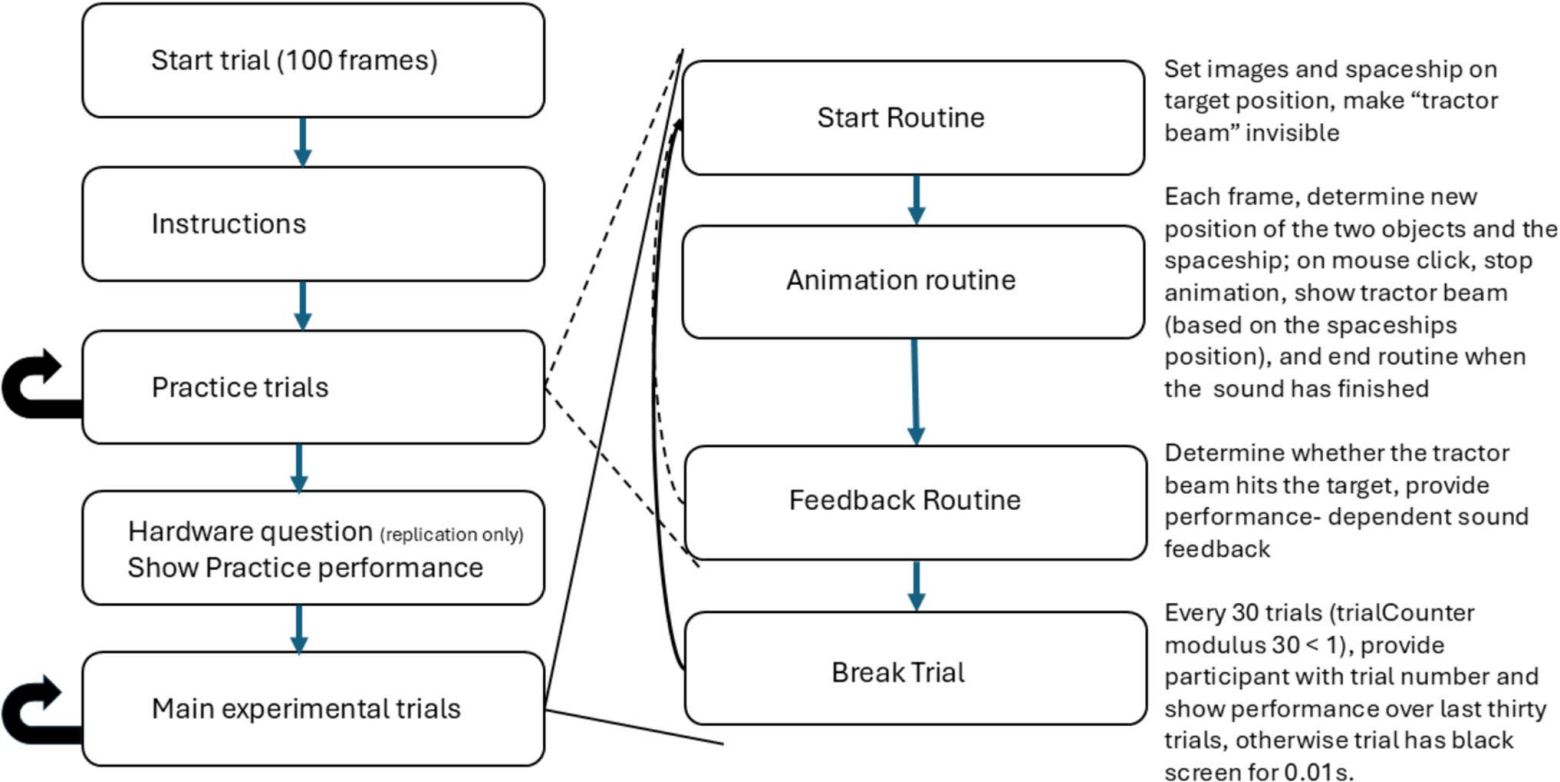
Overview of the structure of the experiment

An important question that was raised by a reviewer with regard to the web-based design is whether there are hardware effects at the user end, especially when they perform the task with an external mouse or using a laptop’s touchpad. However, the initial study did not include a question about that. Therefore, the initial study was replicated adding this question. The results of the two studies will be presented side by side.

## Method

### Participants

Two sets of 40 participants were recruited from Prolific.com for the initial and the replication study with the constraints that they were native speakers of German residing in Germany and were not older than 40 years. In the replication experiment, there were 41 full data sets, since one participant finished the task but then returned the submission. The data set was rejected because it contained too many errors (see below). The sample of the initial study consisted of 32 male and eight female participants, with a mean age of 31.6 years, and the sample of the replication study comprised 26 male and 14 female participants, with a mean age of 30.4 years. The sample size was chosen to be similar to Mitterer and Reinisch (2015). Participants were only allowed to participate on desktop or laptop computers, not on mobile devices or tablets.

### Stimuli

The stimuli were taken from the study of Mitterer and Reinisch (2015). We used the available 34 /h/ and 37 glottal-stop initial items (i.e., orthographically vowel-initial items). For each item, the original materials contain an image for the target, a competitor image that also fits the sentence context, an unrelated distractor, and the spoken sentences with the initial segment deleted or not. The latter predictor was implemented through cross-splicing, so that the sentence with deleted and intact initial segments on the target words were identical save for the initial part of the target word. We also used all filler items with the target present, that is, the audio recordings, the target picture, the competitor picture, and the picture for an unrelated distractor. From the filler items with the target missing from the original study, we generated a short five-trial practice block. Target images were found for these items using an image search, and the competitor was taken from the original study. For the audio stimuli, the onset of the target word and the total duration had been measured and added to the pre-compiled trial orders used to run the study, since the target onset and offset influenced how fast the objects were falling down during the experiment. To run the experiment, 32 different random orders were pre-compiled.

### Procedure

Figure 2 provides an overview of the structure of the experiment, which was implemented using PsychoJS (v. 2024.1.4) using the Builder GUI with extensive use of additional code.^3^ The following presentation will be detailed with a focus on facilitating straightforward replicability of the procedure. Additionally, the experiment is publicly accessible on Pavlovia, and all custom code is provided in an additional document on OSF. This document should make it easier to follow how the different variables are updated over the course of the experiment.

Each session started with a first trial that estimated the frame rate of the set-up of the user and was used to set the speed of the objects falling, since those were adjusted every frame (see Fig. 1). Also, on this trial, one of the 32 pre-compiled random orders was selected and used for this session. After the initial trial, participants were instructed how they were supposed to do the task, including an image similar to Fig. 1. Then they entered the practice phase.

**Fig. 3:**
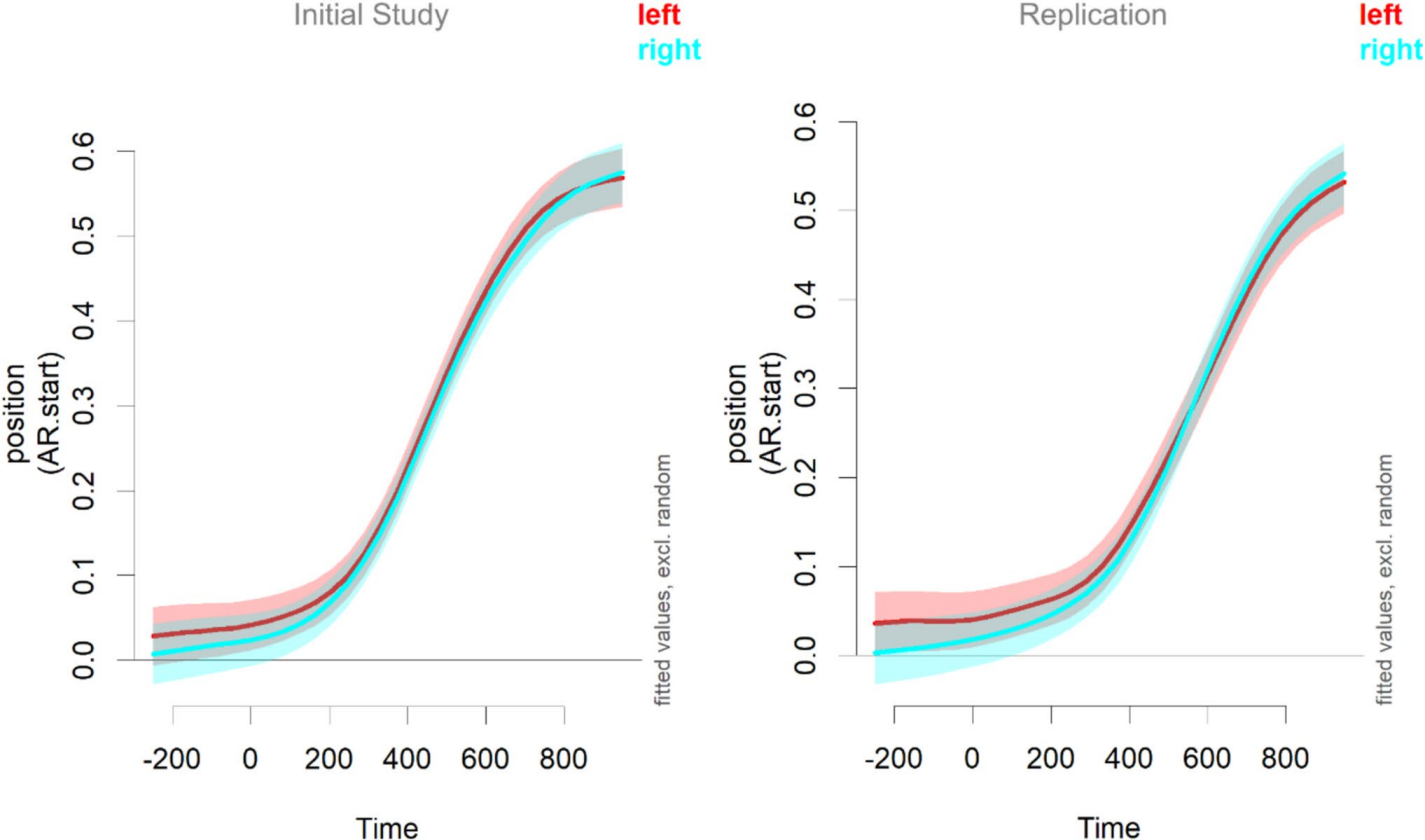
Mouse positions depending on target position (left vs right) in both the initial and the replication study. The shaded areas show confidence intervals based on a GAMM analysis

Each practice trial consisted of three routines in PsychoJS. In the first routine, the three objects (the spaceship and the two images) were shown in their starting positions, and a text appeared asking participants to click on the spaceship to initiate the trial. The text also provided feedback on the trial number, so that participants always knew how much progress they had made (the instructions also mentioned the total number of trials). Figure 3 shows examples of the screen during these three routines in panels A, B, and C for the start routine, the animation routine, and the feedback routine, respectively.

The data object for each trial included the two objects, condition codes, the total duration of the audio file, and the onset of the target word. The falling speed of the objects then was adjusted in this first routine to ensure that they had at least 2 s after the onset of the target word to position the spaceship under the target object. This was necessary, since the target onset differed considerably over trials (from 0.73 s to 3.26 s into the audio file). Note that such an adjustment is quite crucial in order to keep the task uniformly difficult. Otherwise, participants might find some trials impossible to solve with too little time between target onset and the falling objects reaching the lowest possible position, which might lead to demotivation.

When participants clicked the on the spaceship in the initial routine of the trial, the main animation routine started, with the cursor being invisible but the spaceship being set to the position of the mouse in the horizontal direction on each frame refresh. Right at the start of the routine, the sentence started playing. Simultaneously, the positions of the two objects were adjusted on each frame using the pre-established changes per frame in both the *x*- and *y*-direction. The *x*-direction was changed based on a random number drawn on every frame from the interval [0,1]. If that number was smaller than 1/40, the object changed direction. This value was found during development to present a good compromise of objects travelling long enough in one direction but also leading to direction changes unexpectedly before reaching the end of the corridor. As this implies, the *x*-directions were also changed if the objects otherwise would leave their preset corridor on their respective side of the screen. When participants clicked, a “tractor beam” became visible ranging from the current position of the spaceship to near the top of the screen (see Fig. 1). All animations stopped then, and the animation routine ended (with a 200-ms delay making it less jarring) unless the sound was still playing. In that case, the routine ended 200 ms after the sound was finished. If, on a given trial, a participant did not click, the routine was ended when the falling objects reached a vertical position just above the spaceship.

After the animation routine, a feedback routine followed during which the target object was presented at the lowest possible screen location (just above the spaceship) at its latest horizontal position. If the difference between the final location of the spaceship and the target object was less than 5% of the screen width, the “catching” was considered successful, independently of whether the participants made use of the tractor beam or not. Based on that evaluation, a feedback sound was presented to indicate whether the object was considered collected or not, based on typical video game sound (i.e., a bling or a buzzer sound for correct and incorrect trials, respectively). This information was also added to the output data file, along with the final location of both the image and the spaceship.

After five such practice trials, participants were given their percentage of correct responses with the warning that we expected 80% correct responses to consider the task as being done properly. In the replication study, participants were additionally asked whether they moved the spaceship using an external mouse, a touchpad, or a game controller. They were informed that, every 30 trials, they would be provided an overview of their performance.

This was achieved during the main trial sequence by adding one additional routine to the three routines used in the practice trials (see Fig. 2). This routine was unnoticeable for participants on most trials, since it had a short duration (0.01 s) and only consisted of a blank screen. However, when the modulus of the trial number and 30 was zero (i.e., trials 30, 60, and 90), the trial duration was set to a large number (100,000 s or 1,666 minutes^4^), and a feedback text informed participants of their performance over the last 30 trials. The routine ended when the participants clicked with the mouse. The experiment ended after the 96 experimental trials, after which they were redirected back to the Prolific.com site.

## Analysis

### Preprocessing

The results files in csv format contain all the information used to run a given trial plus the information about the mouse on each frame: the timing, an *x*-position, a *y*-position, and whether one of the buttons was pressed. From this record, both the mouse-tracking data and the main dependent variable, the “decision times” were calculated.

In a first step (see readCSV.Rmd/html files on OSF^5^), the different csv files were read in and combined into one large data file, which was then later split into different files for the two types of questions (phonological and semantic processing). During this process, participants with less than 70% correct responses were excluded, since the task is relatively straightforward and such low performance indicates poor overall task performance. Moreover, trials were removed when a mouse click occurred before the onset of the target in the spoken sentence or when the whole trial took longer than 5.2 s, indicating that timing may have been problematic on this trial.

One challenge with online mouse-tracking is that the mouse data are collected at frames and not at given times. This means that the time points at which the mouse locations were measured differ among participants. Therefore, in a next step, the timelines of the different mouse locations were normalized. That is, for a timeline from 0.5 before target onset to 2 s after target onset at 60 Hz (i.e., every 16.67 ms), we found the nearest observed mouse position in time and used it for this time point. In this way, the different sample rates and times over different trials and different host computers were normalized into the same time frame. Note that this method also “pads” the time series of observed mouse locations with the last mouse location if the participant had already ended the trial with a mouse click (see files mousePreprocessing_phon.R and mousePreprocessing_sem.R, available on OSF, which are identical apart from working on the different data sets consisting of trials addressing semantic or phonological processing).

From these time series, a decision time was calculated for each trial (see the markdown files mouseAnalysis_phon/sem). First of all, the mouse coordinates were multiplied by −1 if the target was on the left side. In this way, positive numbers indicate mouse position closer to the target on all trials. Each observed mouse location was categorized as being on the target (if in the target corridor, coded as ‘t’), towards the target (coded as ‘l’, as moving to the left), away from the target (‘r’, as in moving to the right), or stable outside the target corridor (‘s’). These were then concatenated into a string, producing a string with a length of 151 characters (for 151 time points from 0.5 s before target onset until 2 s after target onset at a 60 Hz sample rate). In that string, the latest occurrence of ‘lt’ was found, which indicates the last move into the target corridor. From that point, the string was searched for where the movement towards the target corridor started (i.e., the mouse movement was categorized as towards the target), and the start of that movement was taken as the decision time.

Since, in rare cases, it is possible that the mouse is stable just outside the target corridor and moves into the mouse corridor in one frame, for these cases, the algorithm searched for an ‘st’ sequence, and if one was found and found to be later than the last ‘lt’ sequence, this time point was taken as the time a participant committed to the target object.

It is also important to note that the decision time measures lead to some data loss. In some cases, participants may simply make an initial guess of which object to track, and that guess turns out to be correct. In such cases, they are always on the target, meaning that no decision time can be computed.

### Statistical analysis

The main dependent variable in the preceding mouse-tracking studies (Mitterer et al., 2024; Roettger & Franke, 2019) was the time the participants made the last turn towards the target, coined “decision time”. The preprocessing calculated these decision times, which were then used as the dependent measures and analysed with linear mixed-effects models with participant and item as random effects.

For the trials with deleted segments, the two contrast-coded predictors were whether the initial segment was present (+0.5) or not (−0.5) and whether the initial segment was /h/ (+0.5) or the glottal stop (−0.5). For the trials with semantic cues singling out one object, the only predictor was the semantic predictability of the target (no, −0.5; yes, +0.5). The coding was done so that the level that was “more” (/h/ orthographically coded, glottal stop not; segment present vs absent, target predictable vs not) was always mapped on a positive number. Since we are dealing with a form of reaction time, shorter times indicate better performance, so that we generally expect to see negative regression weights.

All modelling started with a full random effect structure. This led to non-convergence in all cases. To achieve convergence, first of all, correlations between random effects were removed, and if that was not sufficient, the smallest random slope was removed, with the exception that when there was a random slope for an interaction, that slope was removed first.

In addition to the decision times, exploratory analyses were performed on the time-course data of the mouse positions (similar to eye-tracking studies). These analyses looked at the time course from well before target onset (−250 ms) to 950 ms after target onset, when the response curves started to asymptote. For that purpose, we used general additive mixed models (GAMM), following the tutorial by Wieling (2018). The GAMM analyses made use of the function *bam* from the package *mgcv* (Wood, 2023) and the *plot_smooth* and *plot_diff* function from *itsadug* (Rij et al., 2022) for visualization.

For analysis with only one factor, this factor was used as a categorical predictor, with time-varying random intercepts and slope over participants and items. For the analysis of the trials focussing on segment reduction in interaction with segment identity, reduction was used as a factor, and its interaction with segment identity was modelled as a binary smooth that evaluated whether the effect of reduction differed between /h/ and glottal-stop words.

Reaction times were also analysed, but this only included a subset of the participants, because six participants in the initial study and 10 in the replication study did not use mouse clicks to end trials. Note that participants were not required to click. Thus, for a considerable portion of the data set, reaction times are not available. For this analysis, first, an intercept-only model with participant and item as random factors was performed, and reaction times with a residual larger than absolute 3 were removed. This led to roughly normally distributed reaction times, which were then analysed with linear mixed-effect models with the maximally converging random-effect structure.

## Results

### Overall performance

Three participants in the initial and one in the replication study were found to have less than 70% correct responses and were excluded from any data analysis. In what follows, numbers in brackets provide the numbers for the replication study. For the remaining participants, mean accuracy was 92% (93%), with the least accurate participants scoring 74% (79%) correct. Given that this well above the threshold of 70%, this indicates that this threshold may reveal participants with a low commitment to the task. Similar numbers were observed in Mitterer et al. (2024).

Fifteen (13) and 16 (13) trials were removed for overly fast or slow reaction times, respectively (see Method for the criterions). This represents about 0.9% of the data. Another 102 (139) trials, which is about 3.2% (3.9%) of the remaining data, had to be rejected because the mouse position was always on the target.

It is also worth mentioning that six (nine) participants did not use the function of the tractor beam but rather waited for the objects to fall onto the spaceship. We evaluated whether these participants (which we coin “non-clickers”) were using a different task setting and only moved the spaceship rather late towards the target object relative to participants who used the mouse click to end the trials as soon as possible. This was not the case; while these participants had slower decision times than the other 31 (31) participants, Table 1 shows that both groups demonstrate the same directional effects to the experimental manipulations. To further investigate the strategies of these two groups, the length of their movement pattern was calculated, that is, how often on each trial the participant changed their movement direction. While, in the initial study, the non-clickers did fewer movements than the clickers (around 7 vs around 10.5 per trial^6^); the opposite was observed in the replication (9.84 changes on average for the non-clickers compared to 8.82 for the clickers). This shows that the non-clickers did not simply leave the spaceship in the centre of the screen to make only one move towards the target.

**Table 1:** Decision times in milliseconds for participants who use the mouse click to end trials early (“clickers”) and those who waited for the object to fall onto the spaceship (“non-clickers”) for the initial and replication study

		Clickers		Non-clickers	
		Initial	Replication	Initial	Replication
		*n* = 31	*n* = 31	*n* = 6	*n* = 9
Semantic trial	Similar competitor	577	465	600	627
	Different competitor	454	300	469	597
Phonological trials	Segment present	547	431	594	620
	Segment deleted	594	475	735	667

**Fig. 4:**
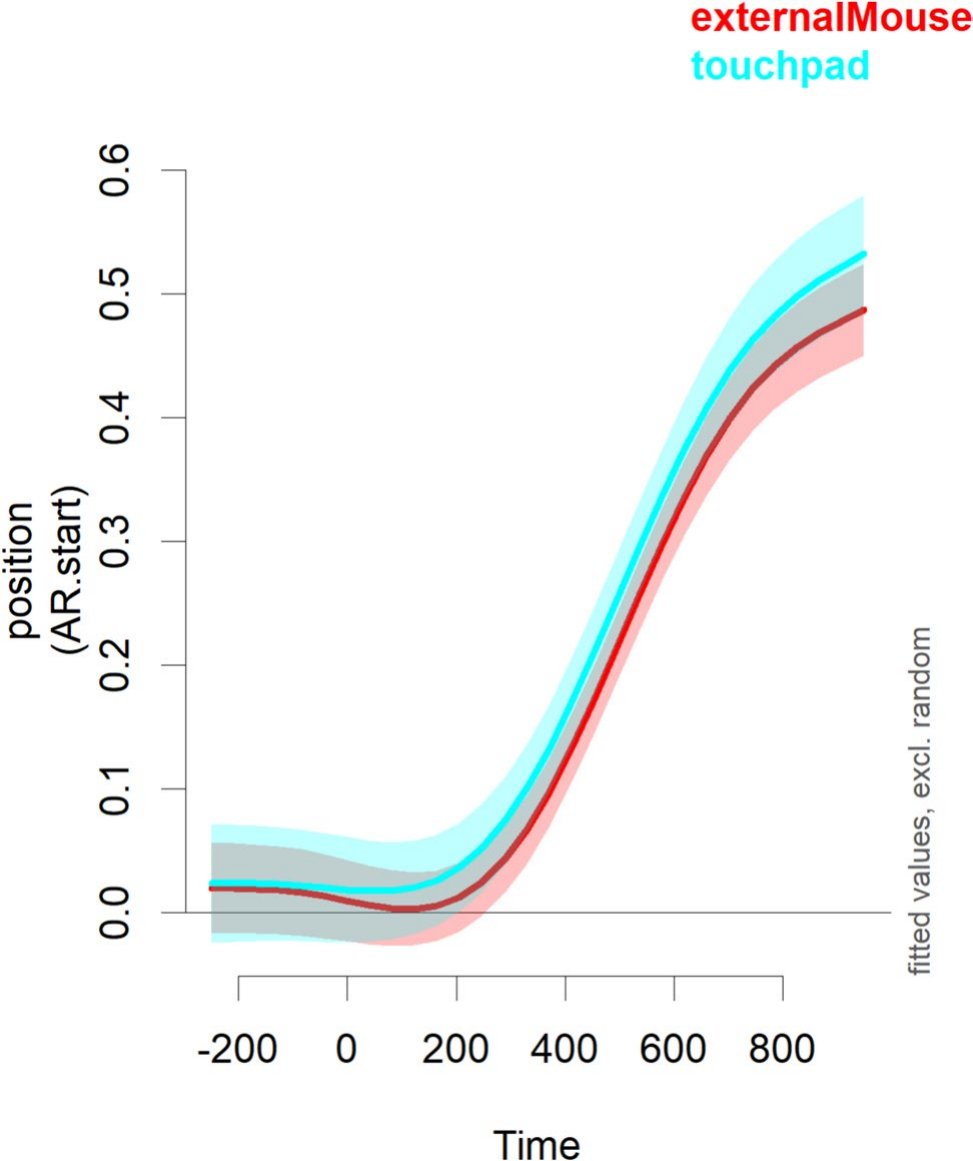
Mouse positions depending on response device (data only available for the replication study)

It was also investigated whether there is a strong effect of target position, given that Spivey et al. found a preference for the left side overall (see their Fig. 1). In eye-tracking studies, it can be observed that participants (from a left-to-right orthography, as in the current study) have a preference to first look at the upper left corner of the screen (see, e.g., Nixon et al., 2016). Figure 4 shows the distance between mouse and target position for trials in which the target is on the left compared to trials in which the target is on the right. There is a small difference, especially in the earlier time window. However, a GAMM analysis with target position as a factor showed no significant differences at any time for either study, as the confidence intervals in Fig. 3 based on the GAMM analysis^7^ show.

Reaction times were about 20 ms faster for targets on the left than on the right in both studies (in the initial study, 1,123 ms vs 1,144 ms; in the replication study, 1,002 ms vs 1,020 ms). This effect was tested using a linear mixed-effects model and was only significant in the initial study (*t*(2,162) = 2.73, *p* = 0.006) but not in the replication study (*t*(2,285) = 0.879, *p* = 0.379). Decision times were very similar for both sides and even reversed direction, with a (non-significant, *t*(38) = −0.63, *p* = 0.535) advantage for the right side in the initial study (left = 568 ms; right = 566 ms) and a (non-significant, *t*(39) = 0.48, *p* = 0.633) advantage for the left side in the replication study (left = 480 ms; right = 483 ms).

### Effects of response device

In the replication study, participants indicated whether they used an external mouse, a touchpad, or a game controller. The latter was not the case for any of the participants. Thirteen used a touchpad, while 27 used an external mouse. Reaction times (for 31 participants who provided reaction times) showed a relatively large difference of about 150 ms (957 ms with external mouse and 1,100 ms with a touchpad). However, in a linear-mixed effects model, this effect was only marginally significant (*t*(25) = −2.05, *p* = 0.051), indicating strong differences in baseline reaction times overall between participants. Figure 4 shows the time course with confidence intervals based on the GAMM,^8^ which indicates that the touchpad users were slightly faster in converging on the target. The GAMM analysis indicates a significant difference between touchpad users and mouse users of between 441 and 538 ms after target onset. However, a comparison to a model without the effect of response device indicated no preference for the more complex model (χ^2^(3) = 5.94, *p* = 0.114). This indicates that effects of response device are small. Decision times were quite similar with an external mouse (485 ms) and a touchpad (472 ms), and a linear mixed-effects model indicated that this difference was not significant (*t*(38) = 0.17, *p* = 0.866).

### Lexical access and reduced word onsets

**Fig. 5:**
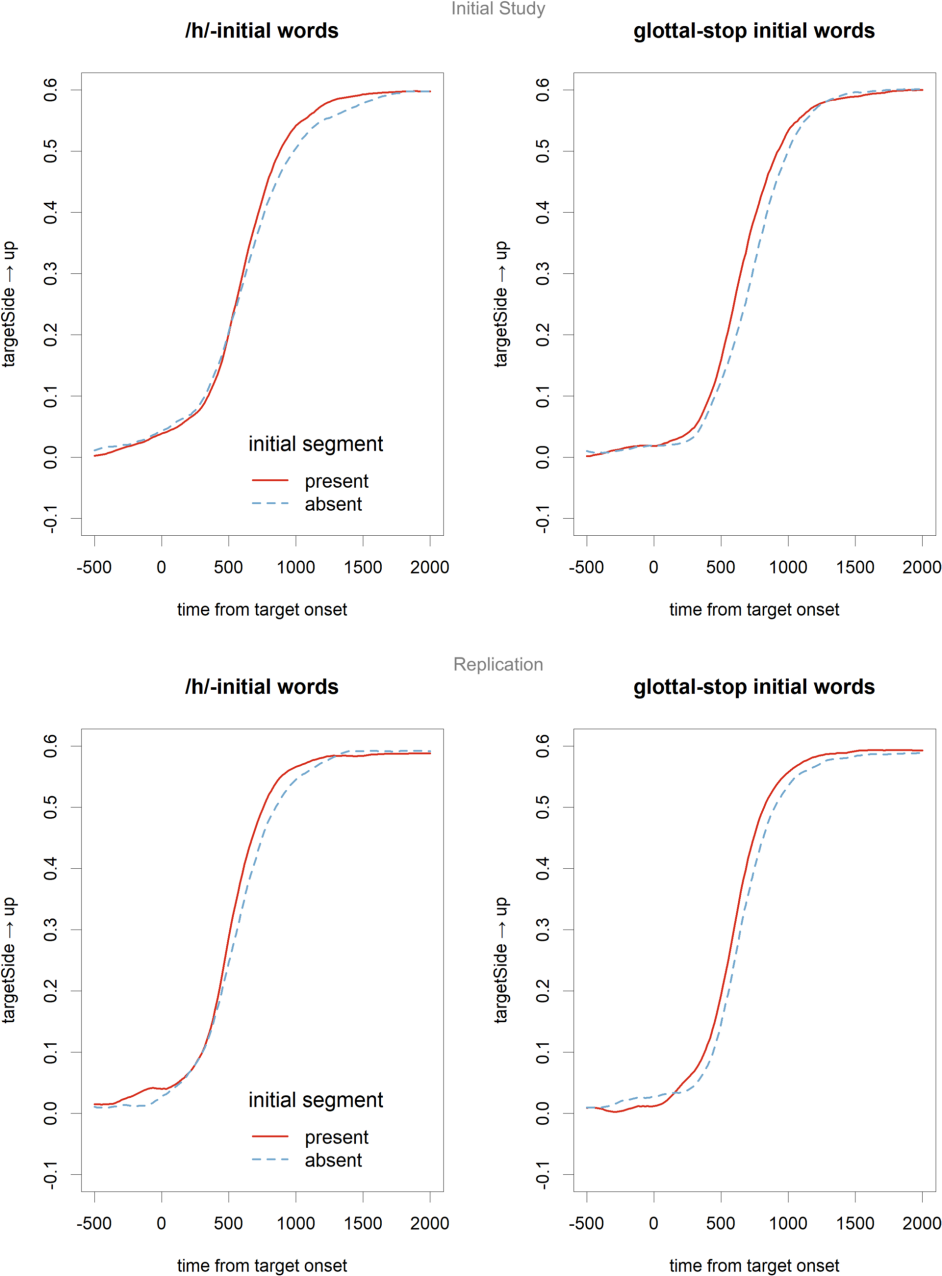
Mouse positions for the trials focussing on lexical access with phonological reductions. The upper two panels show the data from the initial study and the lower from the replication study

Figure 5 shows the position of the mouse relative to the onset time of the target in the spoken sentence. For both glottal stop- and /h/-initial words, there is a small reduction cost visible. These data were analysed with a GAMM^9^. We followed the example of Wieling (2018) and modelled the effect of reduction as factor, and the effect of segment and the interaction as a binary smooth, represented by the terms “s(Time, by = isH)” and “s(Time, by = HisReduced)” in the formula below. Random effects included participants and items, and a time-varying random intercept and random slope for reduction. The analyses indicated a significant effect of reduction between 598 and 817 ms after word onset in the initial study and between 610 and 695 ms in the replication study. However, in both cases, the binary smooth for an interaction of reduction and segment was not significant (initial: *F*(3.7,3.9) = 0.374, *p* = 0.779; replication: *F*(2.17, 2.19) = 0.133, *p* = 0.879).

The analysis of reaction times, which excludes participants who did not use mouse clicks, showed a consistent effect of reduction in the initial experiment (from 1,087 ms to 1,098 ms for /h/-words and from 1,135 to 1,178 ms for glottal-stop words) and the replication (from 956 ms to 1,011 ms for /h/-words and from 1,016 ms to 1,033 ms for glottal-stop words). Linear mixed-effect models show a significant effect of reduction (initial: *t*(18) = −2.88, *p* = 0010; replication: *t*(27) = −2.89, *p* = 0.007). While no other effects were significant, the interaction was marginally significant in the replication, (*t*(65) = −1.97, *p* = 0.053). While this is in the direction of an orthographic effect (stronger effects for orthographically coded /h/), the interaction was in the opposite direction with a stronger effect of reduction for glottal-stop words in the initial study (*t*(65) = 1.06, *p* = 0.29).

**Fig. 6:**
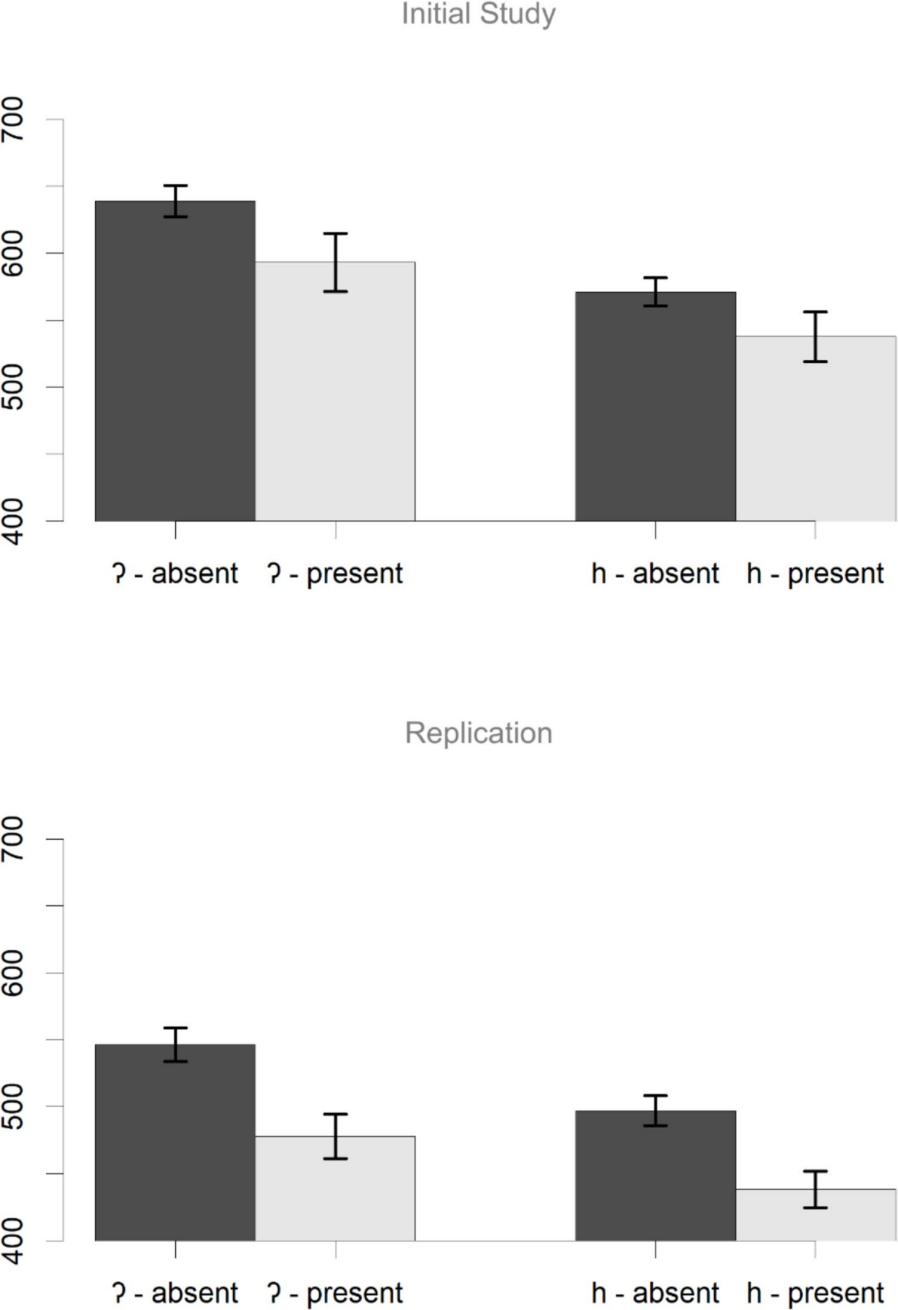
Decision times on trials focussing on lexical access after reduction. The results show a clear effect of reduction that is roughly similar for /h/ and /Ɂ/, despite the difference in orthographic coding. Error bars are confidence intervals based on participant means (Morey, 2008).

More important, however, are the calculated decision times, which also reflect the task given to the participants. Figure 6 shows the mean decision times in the four cells of the design, with error bars based on participant means (Morey, 2008), and Table 2 shows the results of the linear mixed-effects models. The results replicate those of Mitterer and Reinisch (2015), showing a clear effect of reduction that is not modified by the orthographic coding of the deleted segment. From Fig. 6, it might seem surprising that the effect of segment (faster decisions to /h/ initial words) is not significant. Note, however, that this effect is between items, and therefore, the error bars are underestimating the variability here, since they are based on within-participant variability. Note, however, that the main effects of interest here are the effects of reduction, which is defined within participants and within items.

**Table 2:** Results of the linear mixed-effects model for decision times on trials focusing on lexical access

Predictor	*b* (*sd*)	*t* (*df*)	*p*
Initial study			
Intercept	586 (33.8)	17.36 (72)	< .001
cuesC	−69.3 (12.9)	−5.35 (68)	< .001
segmentC	−39.8 (42)	−0.95 (69)	0.347
cuesC:segmentC	4.2 (25.9)	0.16 (68)	0.871
Replication study			
Intercept	489.7 (31.5)	15.55 (85)	< .001
cuesC	−48.4 (14.3)	−3.37 (66)	0.001
segmentC	−60.7 (42.1)	−1.44 (69)	0.153
cuesC:segmentC	−4.3 (28.7)	−0.15 (66)	0.882

### Semantic prediction

**Fig. 7:**
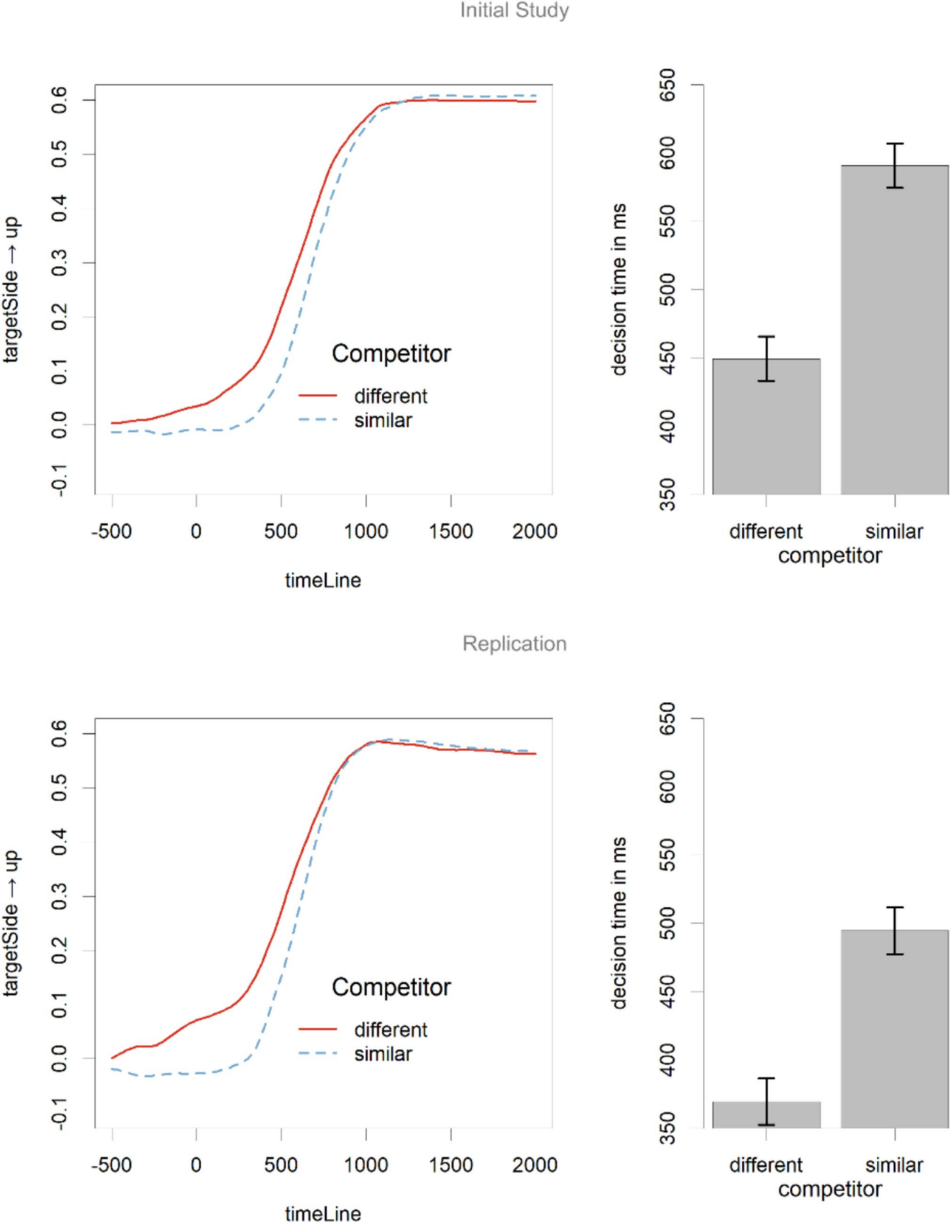
Data from the trials with semantic prediction manipulation. The left panel shows the mouse positions, and the right panel the decision times. The results show a clear effect of semantic predictability. Error bars are based on participant means (Morey, 2008).

Figure 7 shows the mouse data over time and the decision times calculated from them in one figure, given the simpler design with just one condition with two levels. The mouse data over time are presented in the left panel, and the decision times in the right panel.

A GAMM analysis^10^ was performed on the mouse positions over time. The effect of competitor was modelled as a factor, with time-varying random slopes over participant and item. These analyses indicated a significant effect of competitor presence between 40 and 913 ms after target onset in the initial study and between −200 and 700 ms from target onset in the replication study.

The analysis of reaction times, which excludes participants who did not use mouse clicks, showed a consistent effect of semantic competitor presence in the initial experiment (from 1,124 ms without a similar competitor to 1,193 ms with a similar competitor) and the replication (from 968 ms without a similar competitor to 1,050 ms with a similar competitor). Linear mixed-effect models showed that this effect was significant for both the initial study (*t* (21) = −2.93, *p* = 0.008) and the replication study (*t*(23) = −4.17, *p* < .001) Table 3.

**Table 3:** Results of the linear mixed effect model for decision times on trials focusing on semantic predictability

Predictor	*b* (*sd*)	*t* (*df*)	*p*
Initial study			
Intercept	518.2 (36.5)	14.2 (51)	<.001
Competitor similarity	−148.2 (25.3)	−5.86 (22)	<.001
Replication study			
Intercept	430.2 (34.2)	12.57 (53)	<.001
Competitor similarity	−127.1 (29.7)	−4.28 (24)	<.001

## Discussion

The results show that the mouse-tracking paradigm presented here can be used to investigate a wide range of questions in psycholinguistics. Combined with the data by Mitterer et al. (2024), the paradigm seems useful to investigate issues of phonological processing both in the segmental and the supra-segmental domain and for predictive semantic processing.

The results show that the reaction time measures are generally in agreement with the mouse-tracking evidence. Therefore, one might wonder whether it is useful and/or necessary to analyse the mouse movements. As argued in the Introduction, psycholinguistics often makes a distinction between online and offline processing. This distinction is reinforced by dissociation between such measures. For example, Mitterer et al. (2019) found that final lengthening does influence the offline judgement whether a glottal stop in Maltese is lexical or epenthetic, but it did not influence online perception reflected in eye movements in a visual-world paradigm (VWP). The Ganong effect—the finding that listeners are biased to perceive ambiguous speech sounds so that existing words arise—affects perceptual judgements but fails to consistently influence online processing of surrounding segments (McQueen et al., 2009; Pitt & McQueen, 1998). Firestone and Scholl (2016) provide many examples in which experimental manipulations affect perceptual judgements but arguably not perception itself. These considerations indicate that it is useful to find that a late effect in reaction times is also reflected in earlier online measures of cognitive processing.

The combined analyses of time course data and decision times also show that the decision time seems to be a univariate measure that is relatively straightforward to analyse and is relatively robust over the two experiments. The analysis of time course data is clearly possible but becomes quite complex if more than one binary distinction is used (Wieling, 2018). In the current studies, the time course analysis did not reveal additional pattern. It seems, therefore, that the decision times summarize the time course data relatively well and reflect relatively early measures of processing. Having a single data point per trial instead of a full time course also increases the power of any study, since using all timepoints from a given trials leads to the issue of correcting for multiple comparisons, which in turn leads to a reduction in power. These points speak in favour of considering decision times the primary data to analyse in the current paradigm. A possible extension of the data analysis might be the approach by Maldonado et al. (2019), who use linear discriminant analysis (LDA) on the complete data set of the mouse trajectories. Because this requires additional trials with easy and difficult decisions to train the LDA algorithm, it is not possible for the current data set. However, future uses of the paradigm may consider adding such trials to apply this method.

One difference that should be noted between the results obtained here and in Mitterer et al. (2024) is that the decision times obtained here are later than in the earlier study. There is also quite some variability in the studies here; participants were about 80–100 ms faster in the replication study than in the original study. There is no obvious explanation for this; the mean age is very similar for the two studies. The difference between the current studies and that of Mitterer et al. (2024) then is about 100–200 ms. We can observe a similar difference in the displays of the mouse positions over time (see Fig. 4 and 4), in which the inflection towards the target occurs around 350–400 ms after target onset. This is also around 200 ms later than what is typically observed in eye-tracking (Allopenna et al., 1998; Salverda et al., 2014) and also 200 ms later than Mitterer et al. (2024).

This large difference can, however, be explained by some procedural differences between the two studies. In Mitterer et al. (2024), participants heard dialogue that was fixed save for the name of the target object.^11^ That is, participants could anticipate when in the sentence the imperative information would appear. Secondly, Mitterer et al. (2024) used 16 items that were repeated multiple times over the study. Participants also performed a practice block that familiarized them with the items and their depictions before the main experiment. In contrast, in the current study, participants saw each item only once, and the relationship between the visual image and how it was going to be named was not always immediately clear. This is because the set of picturable nouns that start with /h/ or a glottal stop is not large enough. For example, one item was *evening gown* (in German, *Abendkleid*), where participants might also simply have expected *dress* (in German, *Kleid*). These factors probably contributed to the somewhat longer decision in this study in comparison to its predecessor (Mitterer et al., 2024). Therefore, this is an important consideration in setting up future studies with a similar paradigm.

The data also showed that there were no obvious differences between using a mouse or a touchpad. A possible future extension would be to use a touchscreen, but current touchscreens have latency issues, so that it this requires more hardware development (Holden et al., 2020).

It is worthwhile to compare the current paradigm with another option that may give rise to similar data: web-based eye-tracking. At least two recent papers used a web-based eye-tracking task using the VWP, both providing a comparison with a lab-based eye-tracking task, either by replicating an earlier study (Slim & Hartsuiker, 2023) or by running the same study with the same participants on- and offline (Van der Cruyssen et al., 2023). While these studies found a long time delay, this depends on the version of the most prevalent software used for online eye-tracking, WebGazer.js. Yang and Krajbich (2021) found that a small adaptation of the WebGazer.js algorithm led to relatively good timing. However, achieving spatial accuracy in web-based eye-tracking remains a challenge. Yand and Krajbich (2021) used a very thorough calibration and validation procedure, which was repeated three times in total during the experiment. With 13 points, and 3 s of calibration and 2 s of validation for each point, this leads to a relatively long procedure. Comparisons of lab-based eye-tracking with web-based data (Prystauka et al., 2024a) show relatively little time differences but show that target fixations do not quite reach the same ceiling, while distractor and competitor fixations also never reach floor in web-based studies compared to lab-based studies.

This is also evident in a study comparing the quality of web-based eye-tracking with lab-based eye-tracking by Slim et al. (2024). Their data show issues with calibration when using WebGazer by also analysing eye gaze manually from the webcam recordings. Given the similarity in design to the current study, I will focus on the data from their cohort competition task, in which participants had to find a referent for a spoken word (e.g., *milk*) in the presence of a picture that could be described with a similar sounding word (e.g., *mitten*), following the seminal study by Allopenna et al. (1998). They found this well-known competitor effect reliably in web-based eye-tracking and also when annotating eye movements by hand. However, in the WebGazer-based estimation of competitor fixations, the effect was only significant in one of their two studies. This aligns with the finding that small effect sizes may be difficult to replicate in web-based eye-tracking (Prystauka et al., 2024b). Moreover, the fixation proportions for the WebGazer-estimated fixations rarely go above 0.75, and for the distractors, they remain relatively high and rarely fall below 5% (see also Geller et al., 2025). In the hand-annotated data, however, fixations for target and competitor are close to ceiling (1) or floor (0) of the proportion scale, respectively. This suggests issues with the spatial accuracy when using WebGazer.

One speculative interpretation is that this may be due to issues with calibration. In my own experience, even the most cooperative participants sometimes fail to look at a calibration point long enough. In lab-based studies, the experimenter can usually provide feedback to the participant and solve the issue. If that happens in a web-based study, such errors are more difficult to recover from, which would explain why WebGazer.js coordinates fail to reach floor and ceiling in comparison to either lab-based eye-tracking or hand-coded web data, because looks to targets may be coded as looks to other objects, which explains both the lack of ceiling performance for target and the lack of floor performance for competitors and distractors.

These observations show that web-based mouse-tracking can complement web-based eye-tracking. Eye-tracking has the advantage of easily reflecting cognitive processes early and in a natural way (Cooper, 1974; Salverda et al., 2014). The eye-tracking task also only requires participants to pay attention to the stimuli and has been replicated in diverse cultures (Huettig et al., 2011; Mishra et al., 2013). However, small effects are difficult to find without manual annotation, which is costly. Given a trial duration of 6 s, which is typical of VWP studies, the annotation of a trial costs nearly 1 min (based on an estimate of 7 s/min in Slim et al., 2024). For the current study (71 critical trials with 40 participants), this would mean about 40 h of coding time. The current mouse-tracking task may also be preferred when the potential pool of participants is small. A recent study on Spanish–English bilinguals (Geller et al., 2025; this paper is also useful by guiding through the data analysis process) found that around two-thirds of participants failed calibration in an online eye-tracking task. Mouse-tracking, therefore, may be preferred when there are fewer potential participants.^12^

The current task is more gamified than a typical VWP, though it might be possible to further gamify an eye-tracking task, for instance, by moving objects around as in the multi-item localization (MILO) task (Thornton & Horowitz, 2004). The current mouse-tracking task requires some experience with a mouse; however, since attracting participants online at least requires some ability to work with the mouse, this would be a hindrance for any online task that is not mediated by an on-site researcher. The mouse-tracking task has a natural gamified experience which may translate better across cultures. In that context, it is interesting to note that catching games tend to be widespread in cultures around the world (Howard, 1958), so that the current game setting should be easily applied to other cultural settings. These considerations may help to decide between an online eye-tracking or mouse-tracking task. Therefore, the current paradigm holds promise for web-based investigations of language processing and may therefore help to increase the linguistic diversity of psycholinguistic research in concert with eye-tracking measures.

## Data Availability

All code and material is available at https://osf.io/gd6pr/ (data analysis) and https://gitlab.pavlovia.org/holmit/mousetracking_reduction (data gathering).

## Footnotes

1 We would not want websites to put our mouse cursor over an ad and then strongly reduce the maximal speed of that mouse cursor so that we have to look at the ad for a long time.

2 That is not entirely true; the glottal stop is used to mark gender-fair plural forms and is marked in this context (i.e., using *Spion*innen* rather than the generic male plural *Spione* to describe a mixed-gender group of spies). However, for vowel-initial words, the ‘*’ is not used to mark the typical glottal marking of vowel-initial words.

3 The project is available at https://gitlab.pavlovia.org/holmit/mousetracking_reduction

4 In PsychoJS, trials cannot be set to an infinite inherent duration (that is, a trial only ends through some use action) dynamically. Therefore, a trial duration was used that was sufficient even if participants would want to take a coffee break.

5 All files, scripts, and materials are available on OSF: https://osf.io/gd6pr/

6 Note that changes of directions within the target corridor are not included in this number. This means that these participants still moved the mouse around quite a lot on a given trial.

7 The formula for this analysis, with the rho value determined by the auto-correlation value of a model that did not take auto-correlation into account, was as follows:

$$\begin{array}{c}\mathrm{side}.\mathrm{gamm}1 <-\mathrm{ bam}(\mathrm{position }\sim \mathrm{ isTarget }+\mathrm{ s}(\mathrm{Time},\mathrm{ by }=\mathrm{ isTarget}) +\\ \mathrm{ s}(\mathrm{Time},\mathrm{ participant},\mathrm{ by }=\mathrm{ isTarget},\mathrm{ bs }= "\mathrm{fs}",\mathrm{ m}=1) + \\ \begin{array}{c}\mathrm{ s}(\mathrm{Time},\mathrm{ item},\mathrm{ bs }= "\mathrm{fs}",\mathrm{ m}=1),\\ \mathrm{ data}=\mathrm{gamData},\mathrm{ rho }=\mathrm{ myRhoVal},\mathrm{ method}="\mathrm{ML}",\\ \mathrm{ AR}.\mathrm{start }=\mathrm{ gamData\$start}.\mathrm{event},\mathrm{ discrete }=\mathrm{ T})\end{array}\end{array}$$

8 The formula used was as follows:

$$\begin{array}{c}\mathrm{responseDevice}.\mathrm{gamm}1<-\mathrm{bam}(\mathrm{position }\sim \mathrm{ responseDevice }+ \\ \mathrm{s}(\mathrm{Time},\mathrm{ by }=\mathrm{ responseDevice},\mathrm{ k}=5) + \\ \begin{array}{c}\mathrm{s}(\mathrm{Time},\mathrm{ participant},\mathrm{ by }=\mathrm{ responseDevice},\mathrm{ bs }= "\mathrm{fs}",\mathrm{ m}=1,\mathrm{ k }=5) + \\ \mathrm{s}(\mathrm{Time},\mathrm{ item},\mathrm{ bs }= "\mathrm{fs}",\mathrm{ m}=1,\mathrm{ k }=5),\\ \begin{array}{c}\mathrm{data}=\mathrm{gamData},\\ \mathrm{rho }=\mathrm{ myRhoVal},\mathrm{method}="\mathrm{ML}",\\ \mathrm{AR}.\mathrm{start }=\mathrm{ gamData\$start}.\mathrm{event},\mathrm{ discrete }=\mathrm{ T})\end{array}\end{array}\end{array}$$

9 The formula used was as follows:

$$\begin{array}{c}\mathrm{interaction}.\mathrm{gamm }<-\mathrm{ bam}(\mathrm{ position }\sim \mathrm{ realization }+\\ \mathrm{s}(\mathrm{Time},\mathrm{ by }=\mathrm{ realization}) +\mathrm{ s}(\mathrm{Time},\mathrm{ by }=\mathrm{ isH}) +\\ \begin{array}{c}\mathrm{s}(\mathrm{Time},\mathrm{ by }=\mathrm{ HisReduced}) +\\ \mathrm{s}(\mathrm{Time},\mathrm{ participant},\mathrm{ by }=\mathrm{ realization},\mathrm{bs}="\mathrm{fs}") +\\ \begin{array}{c}\mathrm{s}(\mathrm{Time},\mathrm{ item},\mathrm{ by }=\mathrm{ realization},\mathrm{ bs}="\mathrm{fs}"),\mathrm{ rho }= 0.984,\\ \mathrm{data}=\mathrm{gamData},\mathrm{ AR}.\mathrm{start }=\mathrm{ gamData\$start}.\mathrm{event},\mathrm{ nThreads }= 3,\mathrm{ discrete }=\mathrm{ T})\end{array}\end{array}\end{array}$$

10 The formula used was as follows:

$$\begin{array}{c}\mathrm{semantic}.\mathrm{gamm}1 <-\mathrm{ bam}(\mathrm{position }\sim \mathrm{ competitor }+ \\ \mathrm{s}(\mathrm{Time},\mathrm{ by }=\mathrm{ competitor}) +\mathrm{ s}(\mathrm{Time},\mathrm{ participant},\mathrm{ by }=\mathrm{ competitor},\mathrm{ bs }= "\mathrm{fs}",\mathrm{ m}=1) +\\ \begin{array}{c}\mathrm{s}(\mathrm{Time},\mathrm{ item},\mathrm{ bs }= "\mathrm{fs}",\mathrm{ m}=1),\\ \mathrm{data}=\mathrm{gamData},\mathrm{ rho }=\mathrm{ myRhoVal},\mathrm{ method}="\mathrm{ML}",\\ \mathrm{AR}.\mathrm{start }=\mathrm{ gamData\$start}.\mathrm{event},\mathrm{ discrete }=\mathrm{ T})\end{array}\end{array}$$

11 The dialogue consisted of a question (“Have the aliens collect the pear?”) and an answer (“The aliens have collected the pear”) that were constant over trials.

12 To provide an example from my own experience, the pool of Flemish participants on Prolific with German as a second language is relatively small. If the required *N* of analysable data sets is 40, finding them from 150 available participants on Prolific with the required language profile would likely prove difficult.
